# Macrophages in Lung Injury, Repair, and Fibrosis

**DOI:** 10.3390/cells10020436

**Published:** 2021-02-18

**Authors:** Peiyong Cheng, Shuangyan Li, Huaiyong Chen

**Affiliations:** 1 Department of Basic Medicine, Haihe Hospital, Tianjin University, Tianjin 300350, China; peiyong@tju.edu.cn; 2 Department of Basic Medicine, Haihe Clinical College of Tianjin Medical University, Tianjin 300350, China; ushuaiaapril@163.com; 3 Key Research Laboratory for Infectious Disease Prevention for State Administration of Traditional Chinese Medicine, Tianjin Institute of Respiratory Diseases, Tianjin 300350, China; 4 Tianjin Key Laboratory of Lung Regenerative Medicine, Tianjin 300350, China

**Keywords:** macrophage, lung epithelial cells, acute lung injury, lung repair, pulmonary fibrosis

## Abstract

Fibrosis progression in the lung commonly results in impaired functional gas exchange, respiratory failure, or even death. In addition to the aberrant activation and differentiation of lung fibroblasts, persistent alveolar injury and incomplete repair are the driving factors of lung fibrotic response. Macrophages are activated and polarized in response to lipopolysaccharide- or bleomycin-induced lung injury. The classically activated macrophage (M1) and alternatively activated macrophage (M2) have been extensively investigated in lung injury, repair, and fibrosis. In the present review, we summarized the current data on monocyte-derived macrophages that are recruited to the lung, as well as alveolar resident macrophages and their polarization, pyroptosis, and phagocytosis in acute lung injury (ALI). Additionally, we described how macrophages interact with lung epithelial cells during lung repair. Finally, we emphasized the role of macrophage polarization in the pulmonary fibrotic response, and elucidated the potential benefits of targeting macrophage in alleviating pulmonary fibrosis.

## 1. Introduction

In innate immunity, macrophages play a significant role as heterologous phagocytes and express pattern recognition receptors to detect pathogen-associated molecular patterns and damage-associated molecular patterns [1,2]. Macrophages were originally authenticated by Elie Metchnikoff, albeit in a rudimentary manner: in 1883, he reported on the effect of phagocytes in starfish larvae [3]. While the origin of lung macrophages is believed to be complex, these macrophages are formed either by the differentiation of blood-derived monocytes or the proliferation of local macrophages [4].

Tissue-resident macrophages act as the primary and foremost barrier against foreign invaders and coordinate the infiltration of hemameba in innate immunity [5]. Recent lineage-tracing studies have indicated that these macrophages arise from embryonic yolk sac erythromyeloid progenitors; they are capable of self-renewal in large adult tissues at steady-state without the influence of bone marrow hematopoietic stem cells [6]. In the lungs, three different populations of macrophages exist, including airway, alveolar, and interstitial macrophages. Macrophage populations inside the lungs sustain homeostasis via phagocytosing inhaled particulate and foreigner pathogens and inducing cytokine production and antigen presentation, which facilitates the clearance of particulate antigens [7,8]. Alveolar macrophages account for 55% of lung immune cells and are situated on the inner surface of the lung [9]. They participate in the onset of several lung diseases and are crucial for maintaining airway homeostasis. Interstitial macrophages, thus named because they are localized in the interstitial area of the lung, preserve homeostasis and modulate tolerance toward non-threatening antigens [10]. Two independent populations of interstitial macrophages were identified in distinct locations of mouse lung by single-cell mRNA sequencing [11]. Although macrophages play a significant role in defending against invading organisms, their excessive numbers could cause tissue damage [12].

Stimulated by different local environmental factors, macrophages can be divided into two distinct polarization states: classically activated phenotype (M1), and the alternatively activated phenotype (M2) [13]. Evidence has shown that M1 is closely linked to pro-inflammatory responses, while M2 plays a key role in anti-inflammatory reactions [13]. Furthermore, M2 macrophages consist of four subtypes, including M2a, M2b, M2c, and M2d [14]. They are activated by different stimulators. M2a macrophages are elicited mainly by interleukin 4 (IL-4) and IL-13, whereas M2b phenotype is usually stimulated by IL-1 receptor ligands. IL-10 and glucocorticoids promote the synthesis of M2c macrophages, while M2d macrophages are primarily induced by adenosine A_2A_ receptor agonists [14]. M2a cells can promote the production of IL-13, in addition to several other chemokines, such as C-C motif chemokine ligand 17 (CCL17), CCL18, and CCL22. These chemokines are relevant to the activation of Th2 cells and can facilitate eosinophil recruitment into the lungs [15,16]. In addition, M2a cells contribute to the initiation of allergic asthma. By contrast, Treg cells are activated by M2b cells through IL-10, resulting in allergic tolerance and lowered inflammatory responses [17]. Compared to M2a macrophages, M2c macrophages are capable of inducing Tregs and express higher levels of IL-10 and anti-inflammatory cytokines, leading to reduced inflammatory infiltration locally [18]. In addition, these polarized macrophages exhibit plasticity because they can depolarize to M0 macrophages or exhibit the opposite phenotypes by repolarizing (M1 to M2 and vice versa), depending on the types of cytokines present in the specific microenvironment [19].

## 2. Macrophages and Acute Lung Injury

**Figure 1:**
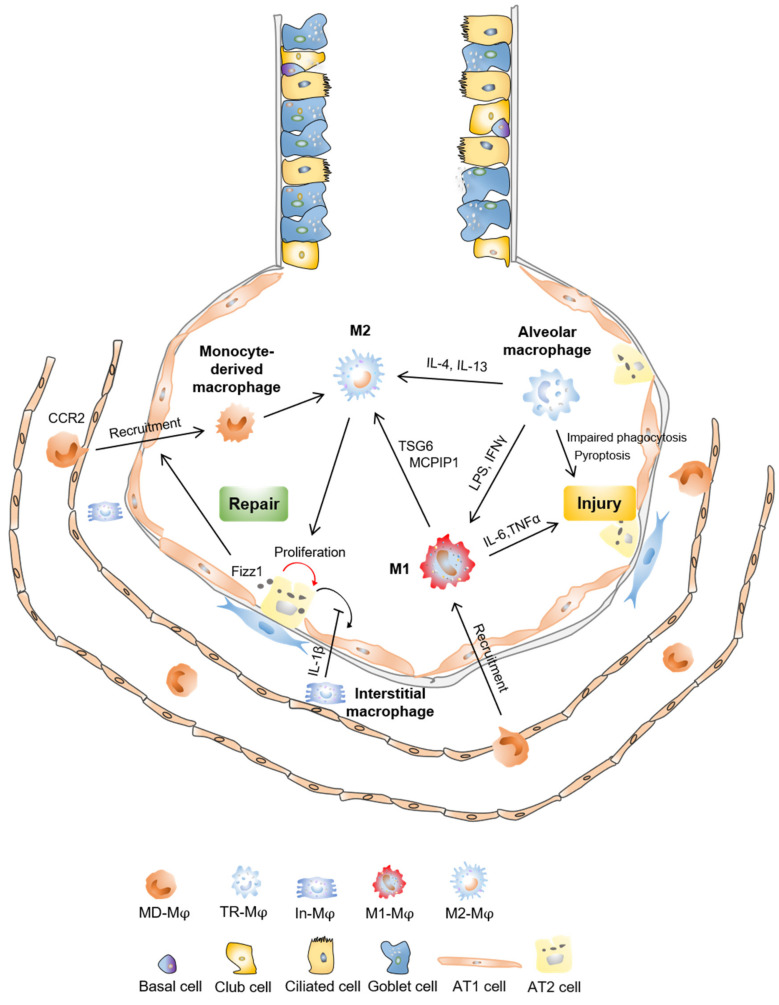
Macrophages in lung injury and repair. Lung tissue resident macrophages and monocyte-derived macrophages can be polarized into classically activated macrophage (M1) phenotype stimulated by LPS and interferon gamma (IFNγ), or into alternatively activated macrophage (M2) phenotype in presence of IL-4 and IL-13. TNFα-stimulated gene-6 (TSG6) and MCP-induced protein 1 (MCPIP1) can convert macrophages from M1 to M2 phenotype. M1 macrophages secrete pro-inflammatory cytokines, such as IL-6, TNFα, leading to enhanced lung injury. Impaired phagocytosis and pyroptosis of alveolar macrophages result in exacerbated lung injury. M2 macrophages produce arginase and contribute to alveolar type 2 cells (AT2) proliferation, resulting in tissue repair after injury. The protein ‘found in inflammatory zone’ (Fizz1) expressed by AT2 cells recruits monocyte-derived macrophages and promotes fibroblast proliferation. Interstitial macrophages derived IL-1β hinders AT1 differentiation.

Inflammation is the host’s necessary defense against immunogenic agents [20]. Inflammation is originally protective, and when homeostasis is restored, the inflammation developed dissipates during repair. However, in case the inflammation is not suitably resolved, pathological fibrosis may develop, ultimately resulting in organ failure [21]. Macrophages execute clearing by phagocytosis; they actively participate in processes such as extracellular matrix remodeling and angiogenesis, as well as inflammation [20]. Acute lung injury (ALI) usually leads to acute respiratory distress syndrome (ARDS), which is the primary cause of death in critical patients [22]. ALI is characterized by leukocyte accumulation, epithelial injury, pulmonary edema, and increased alveolar permeability, as well as diffuse alveolar damage [23]. Macrophage M1 phenotype is involved in the acute phase, while M2 phenotypes are mainly associated with the resolving phases of inflammation in ALI [24]. Macrophage polarization, pyroptosis, and phagocytosis as well as vesicles derived from different cells, are implicated in the inflammation process of ALI (Figure 1).

### 2.1. Macrophage Polarization and ALI

Macrophage polarization can be modulated by multiple factors, including microRNA (miR), proteins, glucocorticoids, and components isolated from herbal extracts. miR is a type of small noncoding RNA, which regulates the stability of mRNAs and the translation process, hence modulating related gene expressions [25]. Several types of miRs have been found to be associated with macrophage polarization and are likely to have promising therapeutic implications in inflammation-related diseases, such as ALI. miR-127 was obviously induced under inflammatory lung conditions [26,27], and lipopolysaccharides (LPS)-induced lung injury is found to deteriorate following the intratracheal administration of miR-127 in mice [28]. Mechanistically, miR-127 represses the expression of target B-cell lymphoma 6 and dual-specificity phosphatase 1 (Dusp 1), followed by enhanced activation of c-Jun N-terminal kinase (JNK), which consequently promotes the activation of M1 pro-inflammatory macrophages [28]. Similar to miR-127, miR-429 attenuates the translation of Dusp 1 and promotes LPS-induced lung injury by boosting alveolar macrophage production of pro-inflammatory cytokines [29]. Metastasis-associated lung adenocarcinoma transcript 1 (Malat1), a long noncoding RNA (lncRNA), has recently demonstrated to promote M1 activation and inhibit IL-4–activated M2 differentiation during bleomycin-induced lung injury [30]. Suppression of lncRNA H19 could reverse toxicant arsenite-induced M2 polarization of macrophages [31]. Apart from noncoding RNAs, a number of proteins and glucocorticoids have been implicated in macrophage polarization and inflammation modulation in ALI [32,33,34]. For example, activated macrophages can generate TNFα-stimulated gene-6 (TSG6). In the LPS-induced ALI model, augmented inflammation and mortality were observed in *TSG6*^-/-^ mice [33]. TSG6 is capable of preventing neutrophil sequestration, decreasing pro-inflammatory mediators, upregulating anti-inflammatory cytokines, and promoting macrophages to convert from M1 to M2 phenotype [33]. MCP-induced protein 1 protects the lungs from ALI by modulating macrophage polarization via suppressing the JNK/c-Myc signal [32]. Methylprednisolone is widely employed in various inflammatory diseases, including ALI, due to its anti-inflammatory properties [35,36,37]. Methylprednisolone promoted M2 polarization instead of M1, thus attenuating tissue damage in ALI. M2, particularly M2c, can induce CD4^+^CD25^+^Fxop3^+^ Tregs and can maintain the immunosuppressive function of Tregs unimpaired, thereby contributing to the resolution of inflammation and tissue repair [34]. Remarkably, with the significant progress achieved in pharmacology research, numerous therapeutic components isolated from herbal extracts demonstrate anti-inflammatory curative effects [38,39]. One of these compounds, Smiglaside A, promotes M2 polarization while inhibiting M1 polarization, likely mediated by the AMP-activated protein kinase (AMPK) and peroxisome proliferator activated receptor gama (PPARγ) signaling pathways [40]. Smiglaside A mitigates LPS-induced lung damage and increases the survival rate of mice. AMPK and PPARγ have been shown to decrease the production of inflammatory mediators, and significantly promote M2 macrophage polarization, thereby ameliorating tissue damage [41,42,43]. Dehydrocostus lactone isolated from herbal extracts suppresses the p38 mitogen-activated protein kinase (MAPK)/MK2 pathway, as well as the production of cytokines, for example IL-6 and IL-1β and pro-inflammatory mediators including inducible nitric oxide synthase (iNOS), resulting in attenuated pathological lung injury [44]. Promoting M2 polarization can also prove beneficial for aseptic lung injury recovery. Clinically, several aseptic conditions such as multiple traumas, burns, acute pancreatitis, and near drowning can lead to ALI, although it is less common than pneumonia and sepsis [45]. Protein kinase B beta (Akt2) is a serine/threonine protein kinase that participates in modulating the macrophage activation phenotype [46,47]. In peritoneal macrophages, the inhibition of the Akt2 isoform suppresses M1 activation while facilitating the M2 phenotype by inducing C/EBPβ and arginase 1 expression [46]. Despite its beneficial effect in aseptic lung injury, regulating macrophage phenotype via Akt2 depletion impairs the ability of macrophages to eliminate live bacteria, thus limiting the use of Akt2 depletion in septic lung injury.

### 2.2. Macrophage Pyroptosis and ALI

Macrophage pyroptosis is associated with the progression of ALI, rather than macrophage polarization. The excessive lung inflammation in ALI is believed to be related to changes in alveolar macrophage activation and death [48,49,50]. Although ALI was believed to be linked to apoptotic cell death, apoptosis elicits a non-inflammatory response, inconsistent with the severe inflammation in ALI [51]. Pyroptosis is caspase-1-dependent programmed cell death, which induces cell swelling and perforates the plasma membrane, leading to potassium efflux, thereby releasing pro-inflammatory substances outside the cells [52,53]. In bronchoalveolar lavage fluid (BALF), alveolar macrophages defend against microbes and airborne particles. They identify pathogen-associated molecular patterns and initiate the innate immune response and host defense mechanisms [48]. Wu showed that caspase-1 was activated in LPS-induced ALI, thereby facilitating alveolar macrophage pyroptosis [54]. Caspase-1 inhibition ameliorates pulmonary tissue damage as well as pulmonary edema in LPS-induced ALI, implying that caspase-1 could be a therapeutic target in the treatment of patients with ALI [54]. In addition to caspase-1, a large body of evidence suggests that the MAPK signal is associated with macrophage pyroptosis [55,56,57]. In the LPS-induced ALI model, p38 MAPK expression and macrophage pyroptosis are upregulated [56]. Blocking the p38 MAPK signaling pathway reorients macrophage death from pro-inflammatory pyroptosis toward non-inflammatory apoptosis [57]. SB203580, a p38 MAPK inhibitor, significantly suppressed macrophage pyroptosis and ameliorated ALI by downregulating NLRP3 inflammasome activation [57]. Thus, inhibition of the p38 MAPK signaling pathway and macrophage pyroptosis may provide a novel immunotherapy strategy for ALI [57].

### 2.3. Macrophage Phagocytosis and ALI

Foreign particles and pathogens engulfed by macrophages comprise an essential step of host innate immunity. Macrophage phagocytic function is essential for the resolution phase of ALI, which can be regulated by transient receptor potential vanilloid 4 (TRPV4), β-adrenergic receptor, α7 nicotinic acetylcholine receptor, and apoptosis inhibitor of macrophage (AIM) [58,59,60,61]. In LPS-induced ALI, the enhancement in macrophage phagocytic function is closely related to TRPV4, as a mechanosensitive ion channel. When extracellular matrix stiffness is altered, TRPV4 can be triggered in conjunction with LPS-induced signals, hence accelerating the rate of macrophage phagocytosis, followed by resolution of lung injury [58]. In an *Escherichia coli*-induced ALI mouse model, human mesenchymal stromal cells (hMSCs) reduce the bacterial burden in the lung and raise the animal survival rate, potentially by boosting macrophage phagocytosis [62]. Catecholamines enhance macrophage phagocytosis of fluorescent *E. coli* bioparticles through β-adrenergic receptor activation, resulting in decreased inflammation, and may be beneficial for ALI resolution [59]. GTS-21 (3-(2,4 dimethoxybenzylidene)-anabaseine dihydrochloride), an α7 nicotinic acetylcholine receptor agonist, can enhance macrophage phagocytosis, contributing to bacterial clearance and decreased acute lung injury induced by *Pseudomonas aeruginosa* [60]. Apart from pathogens, the elimination of apoptotic neutrophils is significant to the resolution of lung inflammation and injury. AIM can prevent macrophages from phagocytosing apoptotic neutrophils, accompanied by exacerbated histopathological damage and inflammation in the lungs. These experimental results further indicate that AIM orchestrates the recovery process of inflammation by altering lipid metabolism [61].

Vesicles derived from different cells also participate in the pathophysiology of ALI. Microvesicles (MVs) are important for intercellular communication, carrying various molecular cargo, such as receptors, proteins, and nucleic acids [63,64,65]. MVs containing TNF, derived from alveolar macrophages, potentially initiate inflammation in vivo and contribute to ALI. These data show that in the pathophysiology of ALI, MVs act as key components and promising therapeutic targets [66]. MSCs are a promising cell-based therapeutic method for ARDS [67]. MSC-derived EVs can transfer mitochondria to human macrophages, resulting in enhanced phagocytosis and decreased pro-inflammatory cytokine secretion [68].

## 3. Macrophages and Lung Repair

The lungs are frequently exposed to microbes and pollutants, thus establishing environmental adaptation that sustains immune tolerance, alleviates tissue injury, and secures gas exchange [7]. Given the clonal analysis and organoid culture together with lineage tracing mouse models, an increasing body of evidence has shown that there exist heterogeneous and complex resident epithelial progenitor or stem cells in the lung [69,70,71,72,73]. They generally divide rarely and are quiescent but proliferate and differentiate rapidly to accelerate the restoration of the surrounding epithelium since the lung is injured [74]. Thus, homeostasis of the lung epithelium is preserved via the endogenous stem/progenitor cells at steady state or after lung injury [75,76,77]. The number of alveolar macrophages was significantly increased in a mouse model of pneumonectomy, suggesting that alveolar macrophages may contribute to secondary alveologenesis [78]. During postnatal development, alveolarization was shown to be associated with the abundance and polarization of macrophages [79].

### 3.1. Macrophages Interact with Epithelial Cells in Lung Repair

Coordinated efforts between epithelia and macrophages are considered indispensable for wound healing (Figure 1), whereas the macrophage-derived molecules that are reliable for repair are barely defined [80,81]. In response to epithelial injury, recruited and resident macrophages drive tissue repair [7]. For instance, after infectious or chemical lung injury, alveolar macrophages may promote epithelial proliferation by producing Wnt ligands [80]. Macrophages could directly boost epithelial proliferation in the absence of other cell types within a co-culture system, including alveolar type 2 cells (AT2) [80]. An in vitro co-culture experiment indicated that macrophages enhance AT2 cell self-renewal and survival [82]. Recent evidence indicates that circulating monocytes rarely promote resident macrophage proliferation at steady state but replace macrophages during inflammation [83,84,85]. Disruptions in the macrophage function could lead to abnormal repairs, as the uncontrolled generation of growth factors and inflammatory mediators, deficient production of anti-inflammatory macrophages, or invalid communication among macrophages and epithelial cells, tissue stem or progenitor cells, and fibroblasts, all contribute to persistent injury, leading to the development of pathological fibrosis [21]. Interestingly, interstitial macrophage-derived IL-1β hinders AT1 differentiation, causing impaired alveolar regeneration and aberrant accumulation of damage-associated type of AT2 cells [86]. Macrophages also induced epithelia to proliferate by trefoil factor 2 generation, a cytokine formerly considered to be released only from impaired epithelia. Thus, lung macrophages may serve as a crucial ancillary source of this pivotal reparative cytokine [80].

Adoptive transfer studies and genetic loss of function in mice indicated that bone marrow-derived monocytes migrate to the lung by a CCL2/C-C motif chemokine receptor 2 (CCR2) axis, which is necessary for AT2 cell proliferation [82]. Mice lacking CCR2 were shown to lose the ability to recruit monocytes in the lung, and display decreased stimulation of AT2 cells and eventually damage compensatory lung regeneration post partial pneumonectomy [82]. Recruited monocytes enhance the generation of the M2-like phenotype of macrophages inside the repairing lung through modulation of the microenvironment [82]. Although it remains unclear whether the recruited and resident macrophages have completely distinct or identical roles in lung regeneration, the findings are specifically interesting to the field since they identify reparative cells that contribute to lung regeneration [87].

### 3.2. Macrophage-Derived Cytokines in Lung Repair

In the lung, if the injury persists, pro-inflammatory signals will continue to be transmitted, and the epithelial cells destroyed by inflammation and infection will be further damaged. Thus, the repair process can be considered an essential part of the settlement of inflammation [88]. After the primary inflammatory phase subsides, macrophages present a wound-healing phenotype by the generation of growth factors, including platelet-derived growth factor, vascular endothelial growth factor alpha, and insulin-like growth factor 1 (IGF-1), which promote blood vessel development and cellular proliferation [89,90,91]. The intricate relationship between resolution of inflammation and repair has been demonstrated by several macrophage-derived cytokines, for example epidermal growth factor-like growth factor amphiregulin [92]. A direct action of amphiregulin in recovering the lung function was originally demonstrated in asthma patients and chronic obstructive pulmonary disease patients; influenza virus-infected mice enhanced amphiregulin expression in the lungs and sputum of such patients [93,94,95]. Airway macrophages produce amphiregulin in response to lipopolysaccharide [96]. In addition, alveolar macrophages generate amphiregulin to offset the epithelial injury induced by infection [88]. Additionally, arginase translates arginine to ornithine, which acts as a precursor for hydroxyproline or proline synthesis, which are the main constituents of collagen [97]. Ornithine enters the biosynthetic pathway of polyamines, which are necessary for cellular proliferation [98]. Moreover, arginase activity controls the inflammatory response because arginase competes with iNOS for their mutual substrate, arginine [97].

### 3.3. Cytokines That Regulate Macrophages in Lung Repair

In experimental helminth infection model, IL-4 receptor signaling was shown to increase IL-10 and IGF-1 expression and stimulate M2 macrophage development, which facilitated the resolution of lung damage [99]. Surfactant protein A was shown to promote IL-4-dependent proliferation and activation of alveolar macrophages, thereby alleviating lung injury after parasite infection [100]. IL-4 expedites resolution of lipopolysaccharide- or *Pseudomonas*-induced ALI and lung repair by reprogramming macrophages to the M2 phenotype [101]. Th2 cells and group 2 innate lymphoid cells predominantly secrete IL-13 and IL-4, which enhance tissue repair and promote the initiation of matrix synthesis via activated macrophages [102,103]. The group 2 innate lymphoid cells generate IL-13, which has been shown to promote lung regeneration [82]. This cytokine modulates the M2-like biochemical functions and enhances their polarization, including collagen synthesis, refactoring of the extracellular matrix, and anti-inflammation [104,105,106,107]. IL-6 is conducive for lung repair following influenza-induced pulmonary injury by promoting the recruitment of macrophages to the lungs and accelerating the rate of phagocytosis of viruses via macrophages [108].

## 4. Macrophages and Lung Fibrosis

**Figure 2:**
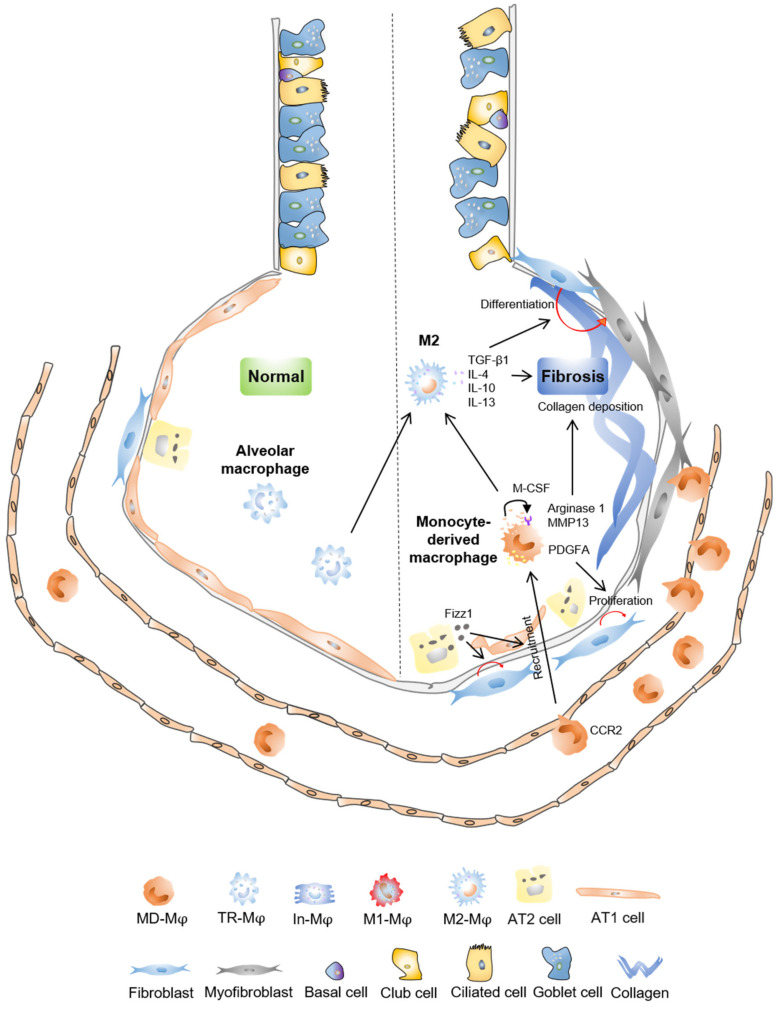
Macrophage in lung fibrosis. Fizz1 expressed by AT2 cells recruits monocyte-derived macrophages and promotes fibroblast activation and proliferation in the lung. Monocyte-derived macrophages secrete macrophage colony-stimulating factor (M-CSF) in an autocrine manner for self-maintenance, and produce platelet-derived growth factor subunit A (PDGFA), arginase 1, matrix metallopeptidase 13 (MMP13) to promote fibrotic process. Both alveolar macrophages and monocyte-derived macrophages can be polarized into M2 phenotype. M2 macrophages produce TGF-β1, inducing the differentiation of fibroblasts into myofibroblasts. Overexpression of IL-4 and IL-10 derived from M2 macrophages also contributes to lung fibrosis.

Idiopathic pulmonary fibrosis (IPF) is a chronic and progressive disease with no effective treatments. Increased myofibroblasts, deposition of collagen, and alveolar epithelial injury are characteristics of IPF and bleomycin-induced fibrosis in animals, resulting in impaired functional gas exchange, respiratory failure, and even death [109,110,111,112]. M2 macrophage, rather M1 phenotype, is involved in fibrotic progress in the lung (Figure 2).

### 4.1. Macrophage Apoptosis in Lung Fibrosis

Macrophage apoptosis is related to pathological fibrosis. Ehab et al. focused their research on glucose-regulated protein 78 (GRP78), which is a primary unfolded protein response regulator, and determined that Grp78^+/−^ mice are remarkably protected against pulmonary fibrosis induced by bleomycin [113]. They further demonstrated that interfering with *Grp78* can upregulate *Chop* and facilitate macrophage apoptosis, leading to reduced lung fibrosis induced by bleomycin. Similarly, high levels of active caspase-8 were observed to induce programmed cell death; furthermore, cellular FADD-like IL-1β–converting enzyme–inhibitory protein (c-FLIP) limits caspase-8 activation, thus its deletion contributes to macrophage death [114,115,116,117,118]. In a bleomycin-induced fibrotic mouse model, RNA-seq revealed that macrophages express profibrotic chemokines abundantly, including CCL2 and CCL24, which recruit fibrocytes and are positively correlated with the progression and severity of fibrosis [119,120]. Loss of c-FLIP in macrophages protects mice from bleomycin-induced fibrosis [121]. Moreover, macrophages derived from BALF of IPF patients also exhibit elevated levels of CCL2 and CCL24 [121]. It seems clear that the elimination of macrophages prevents the development of lung fibrosis. These macrophages expressed markers of both the M1 and M2 phases, but their source was not investigated.

### 4.2. Resident Macrophages and Monocyte-Derived Macrophages in Lung Fibrosis

Depletion of resident macrophages by intratracheally administering liposomal clodronate produces no effect on the fibrosis process [122]. Monocyte-derived macrophages have been proven to contribute to aberrant wound response [123]. Misharin et al. demonstrated that specific genetic deletion of monocyte-derived macrophages after being recruited to the lung can alleviate lung fibrosis [122]. In the lungs of IPF patients, the Wnt/β-catenin is involved in aberrant wound repair, which causes persistent fibrosis [124]. There has been evidence that global loss of low-density lipoprotein receptor-related protein 5 (Lrp5), a Wnt coreceptor, can mitigate pulmonary fibrosis induced by bleomycin [125]. In *Lrp5*^-/-^ mice, gene pathways associated with matrix processing and connective tissue degradation are altered, such as *matrix metallopeptidase 13* (*MMP13)* [125]. In addition, the number of monocyte-derived macrophages in *Lrp5*^-/-^ mice is markedly lower than that in *Lrp5*^+/+^ mice in the fibrotic phase (Day 21 after bleomycin administration), resulting in decreased fibrosis [126]. Through transcriptome analysis, Misharin et al. revealed that several profibrotic genes such as *arginase 1* and *Mmp13* are upregulated in monocyte-derived macrophages compared to the corresponding genes in resident macrophages during the development of lung fibrosis [122]. Additionally, human homologs of these kinds of profibrotic genes are also upregulated in alveolar macrophages in IPF [122]. Using *CD11c^Cre^Casp8^flox/flox^* and *LysM^Cre^Casp8^flox/flox^* mice, which induced necroptosis of monocyte-derived macrophages, they found that the severity of fibrosis is attenuated compared to that in *Casp8^flox/flox^* mice [122]. Finally, they proposed that selectively targeting monocyte-derived macrophages may relieve fibrosis severity without adverse consequences related to circulating monocyte depletion. Later, by applying genetic lineage tracing, in situ RNA hybridization, combined with single-cell RNA sequencing, Misharin et al. further demonstrated a similar role of monocyte-derived macrophages in asbestos-induced pulmonary fibrosis [127]. After asbestos administration, monocyte-derived macrophages were localized to fibrotic areas, expressing platelet-derived growth factor subunit A to facilitate fibroblast proliferation, thus aggravating the severity of fibrosis [127]. Epithelial expression of Fizz1 drives the recruitment of monocyte-derived macrophages to the lungs. *Fizz1*-deficient mice are protected from bleomycin-induced fibrosis [128]. Monocyte-derived macrophages are capable of secreting macrophage colony-stimulating factor (M-CSF) in an autocrine manner, and M-CSF/M-CSFR signaling is indispensable for the maintenance of monocyte-derived macrophages [127]. Pharmacological blockade of this pathway by anti-CSF1 antibody or selective inhibitor PLX3397 greatly reduces the quantity of monocyte-derived macrophages and the severity of fibrosis. Therefore, M-CSF/M-CSFR signaling can serve as a promising drug target for fibrosis therapy. A recent study demonstrated that age-related lung fibrosis is associated with the recruitment of fibrogenic macrophages, which is regulated by endothelial protein C receptor suppression in endothelial cells and IL-1α upregulation in activated platelets [129].

### 4.3. Cytokines/Pathways Associated with M2 Polarization in Lung Fibrosis

Generally, M2 macrophages are important regulators of fibrogenesis, which aggravates the progression of pulmonary fibrosis [21,130,131]. Regulators of M2 polarization contribute to fibrotic progression. IL-4 is known to induce macrophage M2 polarization via activating phosphoinositide 3-Kinase (PI3K)/AKT and Janus kinase 1 (JAK1)/signal transducer and activator of transcription 6 (STAT6) [131,132]. Indeed, during bleomycin-induced experimental fibrosis, IL-4-induced macrophage polarization is modulated by Grb2 associated binding protein 1 (Gab1) and Gab2. Gab1 and Gab2 was found to regulate AKT and STAT6, respectively [133]. Similarly, the phosphatase and tensin homologue loss was observed to sustain PI3K activation and promote macrophage M2 phenotype, leading to exacerbation of bleomycin-induced lung fibrosis [134]. Furthermore, tyrosine phosphatase Shp2 inhibits M2 macrophage polarization and prevents bleomycin-induced pulmonary fibrosis by regulating JAK1/STAT6 activity [135]. Although IL-10 is commonly identified as an anti-inflammatory cytokine, mice with IL-10 overexpression developed lung fibrosis, with a dramatic upregulation of the numbers of M2 macrophages not only in the BALF but also in the whole lung tissue [136]. In IPF patients, circulating levels of serum amyloid P (SAP) are significantly reduced compared to non-fibrotic controls [137]. The circulating SAP is positively related to forced vital capacity, indicating a correlation between SAP and the severity of fibrosis. SAP inhibits pulmonary fibrocyte accumulation and collagen deposition. Administration of SAP is able to reduce M2 macrophages and deplete pulmonary macrophages by liposomal clodronate, leading to anti-fibrotic effects. Therefore, SAP seems to exert an anti-fibrotic effect in TGFβ_1_-induced lung fibrosis by regulating macrophage responses [137]. Recombinant TNF-α reduces the pro-fibrotic M2 phenotype and exerts therapeutic effects during bleomycin-induced fibrosis in mice [138]. In addition to cytokines, adenosine is able to polarize macrophages to M2 phenotype [139]. IL-13 and TGF-β1 expression can be induced by IL-33 signaling through ST2 during bleomycin-induced lung fibrosis [140]. IL-33 and IL-13 synergistically induce M2 macrophage polarization, and anti-IL-33 antibody treatment and ST2 deficiency can attenuate bleomycin-mediated pulmonary inflammation and fibrosis [140].

TGF-β1 overexpression results in progressive pulmonary fibrosis, and depletion of M2 macrophages, which also produce TGF-β1, ameliorates the disease [141]. Besides, TGFβ_1_, which is elevated in fibrotic remodeling, can induce multiple responses related to remodeling, such as fibroblast activation, extracellular matrix deposition, and cell death responses. These processes can be observed in the lungs of IPF, asthma, and scleroderma patients [142,143,144]. M2 polarization is accompanied by an increased expression of a number of surface proteins, including CD206 (also known as mannose receptor) [123,145]. The protein ‘found in inflammatory zone’ (Fizz1) is upregulated by IL-4 on macrophages [146]. Fizz1-deficiency prevents bleomycin-induced lung fibrosis by suppressing lung fibroblast activation and monocyte recruitment to the lungs [128].

In addition, myofibroblast-derived lactate promotes expression of the profibrotic genes in macrophages [147]. Adenosine is generated in response to cellular stress and damage, and signals through the adenosine A2b receptor polarizes macrophages to a fibrotic M2 phenotype during bleomycin-induced fibrosis [139]. It becomes clear that metabolic reprograming is involved in macrophage polarization and lung fibrosis [148].

## 5. Conclusions

There is no cure for pulmonary fibrosis, and abnormal lung repair after epithelial injury will also exacerbate pulmonary fibrosis, which is related to the infiltration of macrophages. The role of macrophages in lung injury repair and fibrosis is very complicated. In the lung injury stage, macrophages are polarized into the M1 phenotype under the action of LPS and interferon gamma (IFNγ) and play a pro-inflammatory function (Figure 1). In the lung repair process, macrophages are polarized to the M2 phenotype in presence of IL-4 and IL-13, accelerating the resolution of inflammation. However, M2 macrophages may also secrete too much TGF-β to promote the proliferation and differentiation of lung fibroblasts and aggravate the progression of pulmonary fibrosis (Figure 2). Blocking the recruitment of mononuclear-derived macrophages, promoting the apoptosis of M2 macrophages or inhibiting the polarization of M2 macrophages may be beneficial for the treatment of pulmonary fibrosis. At the same time, the effective and timely repair of lung epithelia could limit inflammation, thus preventing progression of pulmonary fibrosis. However, it is still not fully addressed how macrophages crosstalk with epithelial stem/progenitor cells, and how the polarization of macrophages impact lung epithelial stem/progenitor cell function. In addition, there is relatively little information on cellular communication between macrophage and fibroblast cells, and endothelial cells in the lung at steady state and after lung injury. Although a lot of studies have linked macrophage M2 polarization to lung repair and fibrosis, the role of subtype of M2 macrophages in lung repair and fibrosis is missing. Besides, future studies are also needed to identify subtypes of macrophages that are associated with fibrotic progression, to characterize the metabolic state of macrophages that regulate their functions, to illustrate the dynamic interaction of macrophages with tissue environments at steady state or in response to insults using single RNA sequencing and multi-omics techniques. Targeting the polarization and source of macrophages has potential benefits for post-injury lung repair and the alleviation of lung fibrosis in clinical treatment for patients with IPF, as well as for patients with severe acute respiratory syndrome coronavirus 2 infection or mycobacterium tuberculosis infection, who may also develop lung fibrosis.

## Data Availability

Not applicable.
